# Scurvy in the Great Irish Famine: Evidence of Vitamin C Deficiency From a Mid-19th Century Skeletal Population

**DOI:** 10.1002/ajpa.22066

**Published:** 2012-03-28

**Authors:** Jonny Geber, Eileen Murphy

**Affiliations:** School of Geography, Archaeology, and Palaeoecology, Queen's University BelfastBelfast BT7 1NN, Northern Ireland

**Keywords:** Vitamin C deficiency, Kilkenny, malnutrition, workhouse, foramen rotundum

## Abstract

Scurvy has increasingly been recognized in archaeological populations since the 1980s but this study represents the first examination of the paleopathological findings of scurvy in a known famine population. The Great Famine (1845–1852) was a watershed in Irish history and resulted in the death of one million people and the mass emigration of just as many. It was initiated by a blight which completely wiped out the potato—virtually the only source of food for the poor of Ireland. This led to mass starvation and a widespread occurrence of infectious and metabolic diseases. A recent discovery of 970 human skeletons from mass burials dating to the height of the famine in Kilkenny City (1847–1851) provided an opportunity to study the skeletal manifestations of scurvy—a disease that became widespread at this time due to the sudden lack of Vitamin C which had previously almost exclusively been provided by the potato. A three-scale diagnostic reliance approach has been employed as a statistical aid for diagnosing the disease in the population. A biocultural approach was adopted to enable the findings to be contextualized and the etiology and impact of the disease explored. The results indicate that scurvy indirectly influenced famine-induced mortality. A sex and stature bias is evident among adults in which males and taller individuals displayed statistically significantly higher levels of scorbutic lesions. The findings have also suggested that new bone formation at the foramen rotundum is a diagnostic criterion for the paleopathological identification of scurvy, particularly among juveniles. Am J Phys Anthropol, 148:512–524, 2012. © 2012 Wiley Periodicals, Inc.

A recent general survey of the paleopathological evidence for disease in Britain observed a noteworthy lack of reported cases of scurvy. This was particularly the case for 18th- and 19th-century skeletons, despite the fact that the disease is well documented in the historical records from these centuries (Roberts and Cox,^2003^). This absence is likely to be primarily a reflection of the misunderstanding and misdiagnosis of the skeletal lesions relating to the disease, particularly in research undertaken prior to the 1980s. The full extent and nature of scorbutic lesions in the human skeleton is still not completely understood, but since this time there has been an increasing interest in the investigation of the skeletal manifestations of the disease (e.g., Maat,^1982^, ^2004^; Maat and Uytterschaut,^1984^; Stuart-Macadam,^1989^; Ortner and Ericksen,^1997^; Brickley,^2000^; Ortner et al.,^1999^, ^2001^; Salis et al.,^2005^; Brickley and Ives,^2006^, ^2008^; Mays,^2008^; Walker et al.,^2009^; van der Merwe et al.,^2010^; Brown and Ortner,^2011^).

The discovery in 2005 of a previously unknown mass burial ground dating to the Great Famine (1845–1852) within the grounds of the former union workhouse in Kilkenny City (52°39′N, −7°15′W) in the south-east of Ireland (Fig. 1) (O'Meara,^2006^; Geber,^2012^) provided a unique opportunity to examine the skeletal manifestations of scurvy in a known famine population. Documentary accounts clearly indicate that these people would have lived in a time of great psychological and physiological stress, and one in which Vitamin C deficiency was omnipresent (Curran,^1847^; Carpenter,^1986^; Crawford,^1988^). The aim of the article is to enhance our understanding of the physical impact of famine on this Irish archaeological population through a detailed analysis of the paleopathological evidence for scurvy. A biocultural approach is adopted to enable the findings to be contextualized and the etiology and impact of the disease explored. A statistical analysis of the scorbutic lesions enables the prevalence of the disease to be assessed by age, sex, and stature so that insights can be gained concerning the relationship between scurvy and mortality on different sectors of the population.

**Fig. 1:**
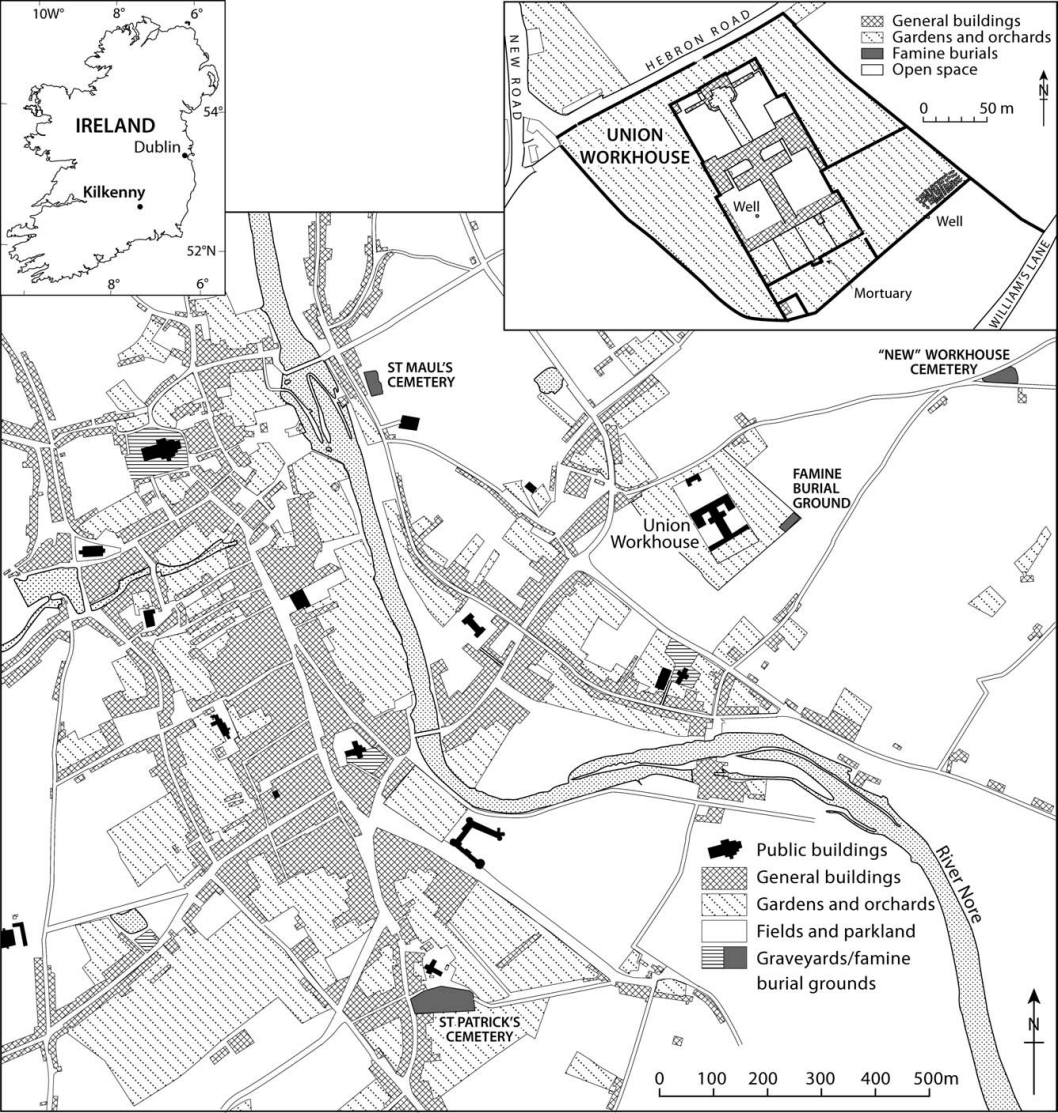
The City of Kilkenny, with the burial grounds used by the Union Workhouse during the Great Famine marked out. Re-drawn from the 1839–1840 Ordnance Survey map.

## Historical background

Largely as a result of economic constraints, the diet of the Irish poor had become increasingly monotonous and was limited to potato and dairy products by the mid-19th century. It has been estimated that the poorest third of the population relied entirely on the potato crop for sustenance at the time (Clarkson and Crawford,^2001^; Ó Gráda,^2006^). A diet consisting mainly of potato and buttermilk was very nutritious, however, and provided more than enough of the required daily intake of protein and vitamins (Crawford,^1988^).

The potato could not be stored from season to season, and certainly not longer than 10 months (Rosen,^1999^), which inevitably resulted in a situation in which many of the poorest members of society starved annually in the summer, between the exhaustion of the old crop and the arrival of the new yield (Woodham-Smith,^1964^; Clarkson and Crawford,^2001^). This vulnerability became critically apparent when the potato blight (*Phytophtora infestans*) reached Ireland in August 1845 and quickly eradicated the bulk of the population's only source of food. The famine of 1845–1852 resulted in one million deaths, and just as many were forced to emigrate. This was in total a population decline of 25% and it resulted in a social and cultural shift which is still evident in Ireland today (Woodham-Smith,^1964^; Boyle and Ó Gráda,^1986^; Kinealy,^2006^; Ó Gráda,^1999^, ^2006^).

## Kilkenny Union Workhouse and the intramural famine mass burial ground

As a measure to deal with the seemingly chronic pauperism in Ireland, a Poor Law was introduced in July 1838. The Poor Law, which was designed to provide a “more effectual Relief of the Destitute Poor in Ireland” (O'Connor,^1995^: 68), was based on the ideological perception that people had a natural passion for idleness, and that disincentives were needed to enable the poor to evade poverty (see de Tocqueville,^1835^). The Poor Law introduced a new workhouse institution to Irish society which, throughout its existence, was genuinely hated. In-door relief in the workhouse was to become the only form of government poor aid for the truly deserving. It was believed that each applicant would have “weighed the ‘pleasures' of staying outside the workhouse with the ‘pain’ of entering it’ (May,^2003^: 8); meaning that only the truly desperate and destitute would accept relief and the able-bodied would be encouraged to find work and improve their situations by themselves. As a consequence, these institutions were built to low standards and inmates were subjected to arduous and repetitive labor. Kilkenny Union Workhouse had an allocated space for 1,300 inmates and was to become the fifth largest workhouse in Ireland when it first opened in April 1842. During the famine it became critically overcrowded with starving people, who in desperation had sought help, and by June 1851 a massive 4,357 people were registered in its books. The gross overcrowding meant that the spread of infectious disease, and consequential mass deaths, resulted in further logistical and economic challenges to the workhouse institution.

No designated burial plot had been assigned to the Kilkenny institution when it was initially established. At first, local cemeteries in Kilkenny City were used, but following the intensification of the crisis these eventually became so critically full that it was necessary to bury the deceased inmates within the grounds of the workhouse itself. Contemporary newspaper articles, and the surviving original minute books from the workhouse, have indirectly revealed that the intramural burial ground was in use between August 1847 and March 1851 (Geber,^2012^). After the famine, there may have been an intentional effort to conceal its presence—the burial ground was deliberately covered with a thick layer of sterile soil, and later used as the workhouse garden, and its location was never marked on any maps. It is a common trait of posttraumatic populations to be reticent in discussing their experiences and this may well have been the case for the survivors living in postfamine Ireland (Ó Gráda,^2001^). By the time of its discovery in 2005, no local traditions existed in relation to the burial ground and, as such, the find was completely unanticipated. The archaeological excavation revealed that the burial ground comprised a minimum of 63 mass burial pits, wherein the deceased had been interred in simple pine coffins stacked one on top of the other.

## Vitamin C deficiency

Scurvy is the consequence of an inadequate intake of Vitamin C (ascorbic acid). The introduction of the Vitamin C-rich potato in the seventeenth century had allowed the population of Ireland to increase to around eight million, the majority of whom were landless peasants. The blight had a devastating impact on the availability of Vitamin C to the poorest members of Irish society who were dependent on the tuber for their survival (Clarkson and Crawford,^2001^). A potato-based diet has a very high Vitamin C content—freshly dug potatoes hold approximately 30 mg of Vitamin C per 100 g of edible matter, which decreases to 8 mg after 8–9 months of storage. Cooked potatoes lose between 20 and 40% of their vitamin content, while an additional 10% is lost in peeled potatoes. The richness of the potato as a source of Vitamin C becomes clear when these values are compared to other foodstuffs such as apples and pears, for example, which only contain 2–5 mg Vitamin C per 100 g edible matter. The levels of Vitamin C in potatoes are more similar to those of citrus fruits, with lemon juice containing approximately 40–50 mg per 100 g of edible matter (Crawford,^1988^). A sufficient intake of Vitamin C is essential for the formation of mature collagen, which in turn is an important protein component in connective tissues such as skin, cartilage, and bone (Stuart-Macadam,^1989^; Hirschmann and Raugi,^1999^). A joint report by the World Health Organization and the Food and Agricultural Organization of the United Nations (2004) recommends a daily intake of 45 mg in adults, although experiments have shown that an absolute minimum requirement is 10 mg or even less (Bartley et al.,^1953^). Males require a slightly higher intake of Vitamin C than females due to metabolic, and possibly hormonal, differences (Basu and Schorah,^1982^; Clarkson and Crawford,^2001^).

The character of the disease is dependent on the age of the affected individual. Infantile scurvy is particularly prevalent between the sixth and twelfth month of age and its main cause in the 19th century was a lack of fresh vegetables in the diet. Scurvy is more likely to appear in infants who are no longer breastfeeding, although a child that is breastfed by a severely malnourished and Vitamin C-deprived mother will, as a consequence, also become deficient (Brickley and Ives,^2008^). Scurvy can also develop in infants who are fed solely on cow's milk (Wing and Brown,^1979^), and the presence of the disease in young infants may reflect the inability of some mothers to lactate due to their own poor health.

Vitamin C can be stored in the human body for a period of four to five months in adults, and it is after depletion for this length of time that scorbutic symptoms appear (Stuart-Macadam,^1989^). The historical record for the Great Irish Famine corroborates this trend, and the first cases of scurvy were noticed by the medical profession at the end of 1845. One of the earliest accounts of the disease was of people in the town of Naas in County Kildare, who in November 1845 were described as suffering from “rose-colored patches,” which would have been due to ecchymoses, and severe pains in their bones and swollen muscles “so acute that the patients [winced] on the slightest pressure” (Crawford,^1988^: 286). This account provides a graphic description of the severe pain suffered by those afflicted with scurvy. Excruciating pain is known to develop early in the disease and often results in pseudo-paralysis. Other clinical consequences include gingivitis, tooth loss, swelling of the lower extremities, and perifollicular hemorrhages (Sullivan,^1903^). Additional symptoms involve alternate feelings of being hot and cold, vertigo, faintness, profuse sweating, hemorrhagic spots in the eyes, xerosis, hyperkeratosis, bent and coiled body hairs, and impaired healing of wounds (Hodges et al.,^1969^; Hirschmann and Raugi,^1999^).

## Skeletal markers of scurvy

The osseous manifestations of scurvy have been comprehensively described in the paleopathological literature (e.g., Maat and Uytterschaut,^1984^; Stuart-Macadam,^1989^; Ortner and Ericksen,^1997^; Ortner et al.,^1999^, ^2001^; Ortner^2003^; Maat,^1982^, ^2004^; Salis et al.,^2005^; Brickley and Ives,^2006^, ^2008^; Mays,^2007^, ^2008^; Brown and Ortner,^2011^). The osseous changes predominantly involve vascular responses to hemorrhages, usually appearing as a result of minor trauma and mechanical strains, which results in porotic and hypertrophic bone formation at the affected areas. Many of these traits are nonspecific, however, and may occur in other metabolic disorders such as anemia and rickets. Other even less specific observable traits include fractures, due to a weakened bone structure, and transverse fractures and dislocations of the osteochondral junction of the ribs (see Brickley and Ives,^2006^). As Vitamin C is required for osteoid formation, skeletal evidence of scurvy is only evident once the vitamin has been re-introduced into the diet after a period of deficiency (Brickley and Ives,^2008^). The lesions are therefore a reflection of the disease during its convalescence stage or after it has been cured. This is an important factor to consider when interpreting its prevalence in skeletal populations since individuals with no osseous lesions may have been severe sufferers who died before any scorbutic lesions had manifested (see Wood et al.,^1992^).

## MATERIAL AND METHODS

The skeletal population from Kilkenny Union Workhouse comprised a minimum of 970 individuals. Two-fifths of the skeletons (*N* = 396) were virtually complete or complete, and more than 80% of the skeleton was present in 68% (*N* = 661) of all analyzed individuals. Neonates and infants represented the least well preserved skeletons.

### Osteological methodology

The skeletons were analyzed macroscopically following recommended standard osteological methodologies (Buikstra and Ubelaker,^1994^; Brickley and McKinley,^2004^). The juveniles were primarily aged on the basis of dental development and eruption following the Bolton Standards by Broadbent et al. (1975), and also through examination of the crown and root formation of deciduous and permanent teeth using the methods presented by Liversidge et al. (1998), Moorrees et al. (1963), and Smith (1991), and through an evaluation of the degree of epiphyseal fusion (Scheuer and Black,^2000^). Age-at-death in adults was estimated primarily from degenerative changes to the pubic symphyses and auricular surfaces of the pelvis (Lovejoy et al.,1985b; Brooks and Suchey,^1990^), the morphology of the sternal ends of the ribs (İşcan et al.,^1984^, ^1985^), and suture obliteration of the cranial vault (Meindl and Lovejoy,^1985^). The age range given to each adult was determined using a multifactorial summary age determination technique (Lovejoy et al.,1985a), weighed against age estimations derived from the pubic symphysis. The mean age-at-death of the population was calculated from the mid-value of the age range estimation in each skeleton. Age groups were assigned based on the recommendations by Falys and Lewis (2011) and Powers (2008)—neonate (less than 1 month); infant (1–12 months); young child (1–5 years); older child (6–12 years); adolescent (13–17 years); young adult (18–25 years); early-middle adult (26–35 years); late-middle adult (36–45 years); older adult (≥ 46 years), and indeterminable adult (> 18 years).

Sex in adult skeletons was determined following the morphological descriptions by Buikstra and Ubelaker (1994), primarily focusing on pelvic traits and secondarily on cranial features. In a few cases (*N* = 14), sex was determined from measurements where mean values for each sex had been determined from discriminatory analysis of osteometrics from postcranial elements derived from morphologically sexed adult skeletons. Living stature was estimated using the regression equations of Trotter and Gleser (1952, 1958), excluding the tibiae for female equations (see Jantz et al.,^1994^), and the bone chosen for assignment of stature in each individual skeleton was chosen in order of best correlation to the results given from the left femur.

### Statistical analyses

Statistical analyses were performed using the SPSS statistical software package (SPSS Release 19.0.0. IBM SPSS Inc., 2000). Pearson's two-tailed correlation tests were employed for assigning adult age-at-death using the multifactorial age determination technique and investigating the relationship between scorbutic skeletal lesions. Independent two-sample *t*-tests were performed to compare mean values between group variables, while a chi-square test was used to compare prevalence rates of scurvy between the sexes, and a two-sample Kolmogorov–Smirnov test was employed to assess differences in age distribution between scorbutic and non-scorbutic skeletons.

### Paleopathological diagnosis of scurvy

Scurvy is most reliably diagnosed by assessing a combination of lesions (Brown and Ortner,^2011^; Ortner et al.,^1999^). Brickley and Ives (2008) presented weighed diagnostic criteria based on clinical features required for a positive paleopathological diagnosis of scurvy. Although these were considered, a systematic diagnostic approach is difficult to apply to partial and fragmented skeletons which are particularly common in archaeological skeletal populations and inevitably results in an under-representation of the prevalence rates of the disease (see Waldron2007a). As the majority of skeletal indicators of scurvy are generally nonspecific, the disease in the Kilkenny Union Workhouse population was therefore assessed and classified as definite, probable, and possible diagnoses with the aid of statistical correlation analyses. This approach enabled a more consistent diagnosis across the entire population, which considers as many diagnostic lesions as possible, even in skeletons that were not anatomically complete. At the same time, it ensured that only variables that were statistically correlated to each other—and therefore less likely to be generally nonspecific—were included in the assessment. Since juveniles and adults generally display different markers of the disease they were assessed separately. Analysis of complete skeletons indicated that all of the lesions were manifested bilaterally, as would be expected in scurvy.

**Fig. 2:**
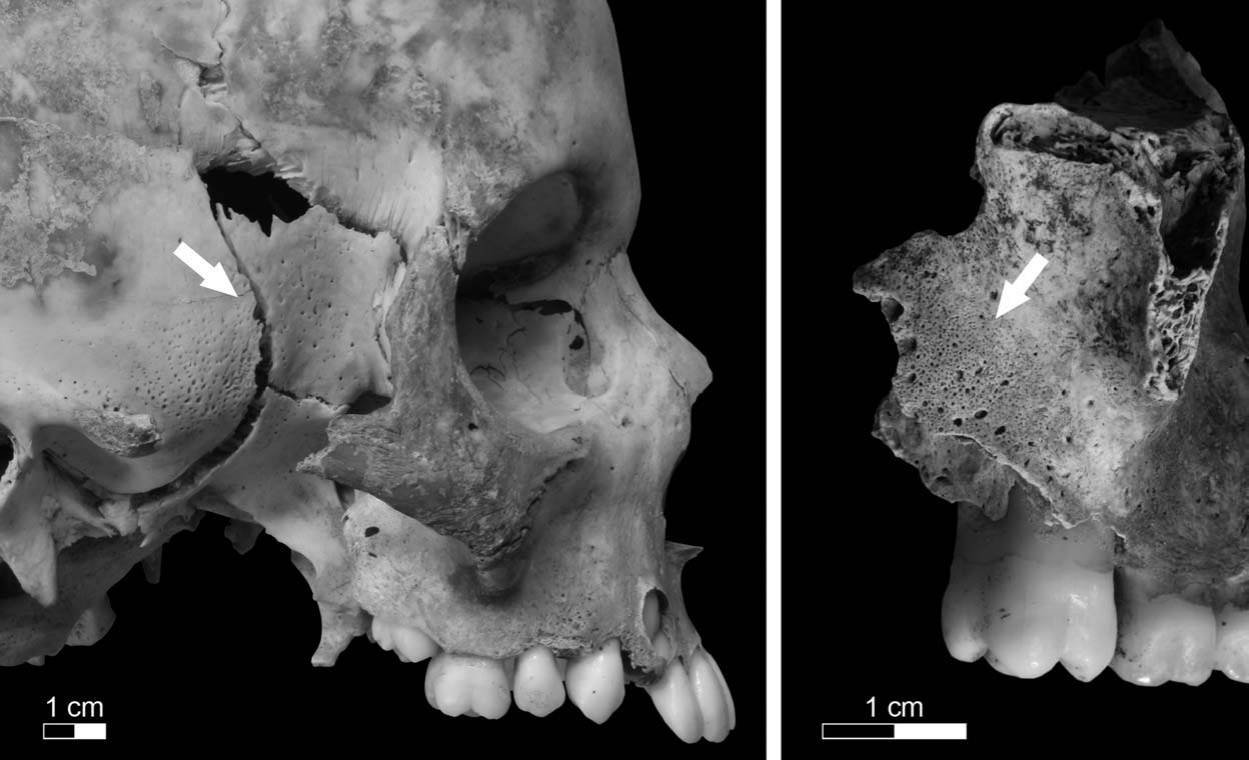
Example of definite diagnostic scorbutic traits—porotic lesions to the greater wing of the sphenoid (10–11 year old child) (left) and porous bone formation on the posterior surface of the maxillary body (9–10 year old child) (right).

A definite diagnosis of scurvy, in both juveniles and adults, was made in skeletons that displayed active porotic lesions of the greater wings of the sphenoid, porous bone formation of the posterior surface of the maxillae (Fig. 2), and porotic lesions on the medial surface of the mandibular ramii. These lesions reflect the involvement of the temporalis musculature, which is used during mastication and movement of the lower jaw, and have previously been described as virtually pathognomonic of scurvy (Brickley and Ives,^2008^; Ortner et al.,^1999^, ^2001^). In addition, definite scurvy was also diagnosed in skeletons with abnormal porosity and plaques of porous new bone formation on the alveolar bone and palatine processes which was clearly not due to generalized periodontal disease. These lesions relate to hemorrhaging around erupting teeth in juveniles and petechial hemorrhage and gingivitis of the gums in adults, all of which are known clinical traits of scurvy (Hodges et al.,^1971^; Weize Prinzo,^1999^).

**Fig. 3:**
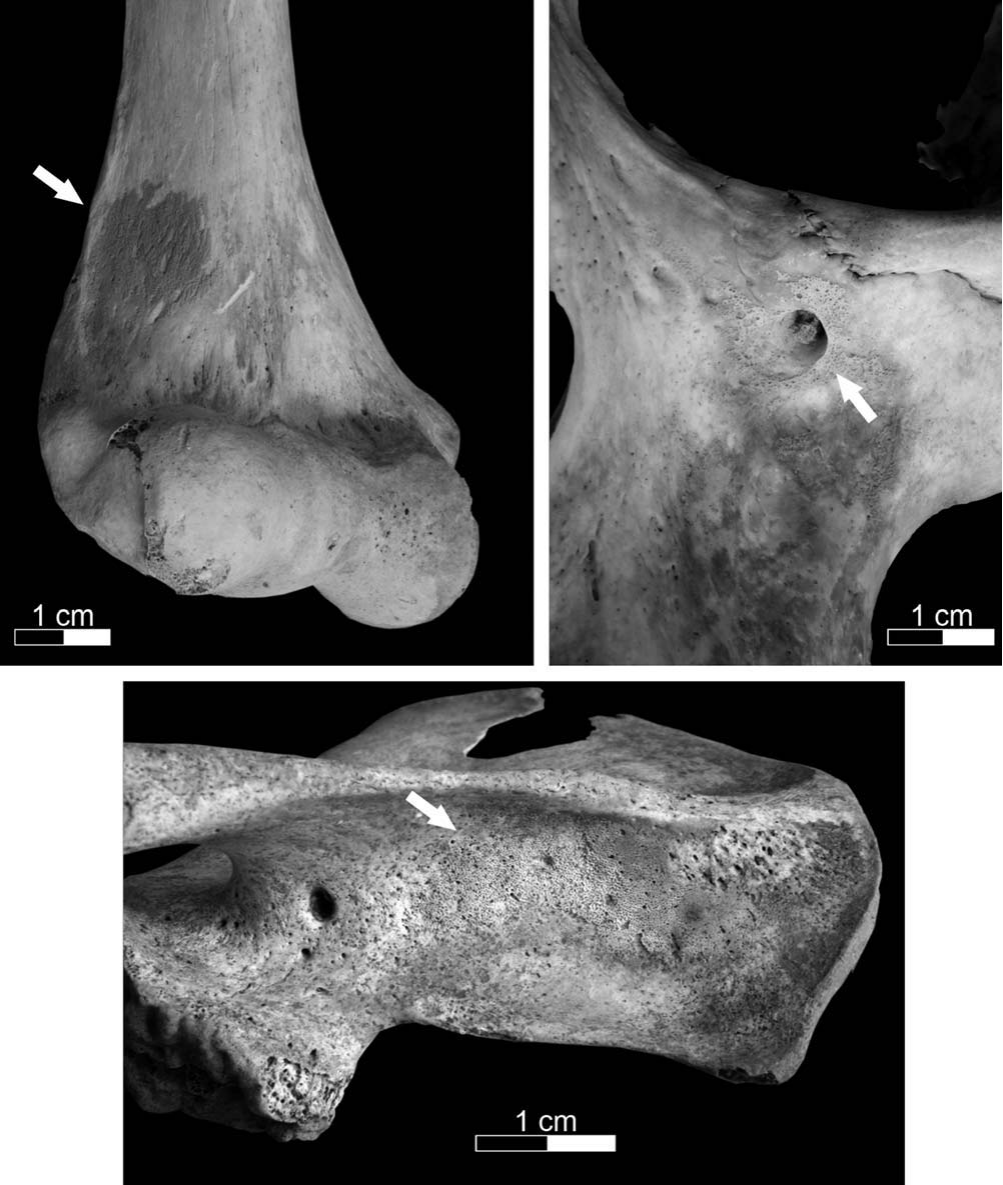
New bone formation on the distal humerus (18–25 year old male) (upper left), the infraorbital foramen (26–35 year old female) (upper right), and the supraspinous area of a right scapula (7–8 year old child) (bottom).

The remaining lesions, which in the literature have been discussed in association with scurvy, were tested in a two-tailed Pearson's correlation test against the definite variables (Supporting Information Tables S1 and S2). Lesions that were positively correlated to at least two definite variables were labeled as indicative traits of scurvy, and when at least two of these indicative variables were present they formed the basis of a probable diagnosis of scurvy. In juveniles, these comprised active endocranial proliferative lesions of the frontal bone, porotic, and hypertrophic lesions of the lesser wings of the sphenoid, porotic lesions on the orbital surfaces of the zygoma, porosity and hyperostosis at the infraorbital foramina, periosteal lesions on the supraspinous area of the scapulae (Fig. 3), and periosteal new bone formation along the linea asperae of the femora and on the diaphyses of the tibiae (Table 1). This pattern follows much of what has been reported in previous studies of juvenile scurvy (e.g., Ortner and Ericksen,^1997^; Ortner et al.,^1999^, ^2001^; Brown and Ortner,^2011^). The correlating indicators in adult skeletons comprised active porous lesions at the infraorbital foramina (see Fig. 3), new bone formation on the radii, ulnae, coxae, tibiae, fibulae and on the diaphysis, and particularly along the linea asperae, of the femora, and in combinations where either all the long bones and the lower limb bones only displayed new bone formation at any location (Table 2). Bilateral periosteal lesions of the lower limb bones, reflecting the formation of ossified hematomas, was described—along with periodontal disease—as possible diagnostic traits of adult scurvy in late 19th-century miners from South Africa by van der Merwe et al. (2010), and were also evident in cases of adult scurvy in 18th- and 19th-century whalers buried at Spitsbergen of the Svalbard Archipelago in the Arctic (Maat and Uytterschaut,^1984^; Maat,^2004^). The correlation between postcranial subperiosteal lesions and cranial scorbutic features in the Kilkenny Union Workhouse is indicative of a similar pattern.

**TABLE 1:** Prevalence rates of scorbutic lesions in juvenile skeletons by age group

	< 1 month		1–12 months		1–5 years		6–12 years		13–17 years		All	
						
	No./Total	%	No./Total	%	No./Total	%	No./Total	%	No./Total	%	No./Total	%
Definite variables												
1. Sphenoid, greater wing	0/18	0.00	4/36	11.11	17/180	9.44	8/116	6.90	2/37	5.41	31/387	8.01
2. Maxilla, posterior	0/15	0.00	0/35	0.00	6/184	3.26	12/133	9.02	4/45	8.89	22/412	5.34
3. Mandible, medial coronoid	0/24	0.00	1/48	2.08	26/216	12.04	17/153	11.11	5/45	11.11	49/486	10.08
4. Palate, process	0/11	0.00	0/29	0.00	8/171	4.68	5/122	4.10	0/42	0.00	13/375	3.47
5. Maxilla, alveolar process	0/14	0.00	0/33	0.00	7/186	3.76	12/132	9.09	3/44	6.82	22/409	5.38
6. Mandible, alveolar process	0/25	0.00	1/52	1.92	14/221	6.33	16/153	10.46	7/45	15.56	38/496	7.66
Indicative variables												
7. Frontal, endocranial	2/20	10.00	1/50	2.00	29/209	13.88	7/140	5.00	0/43	0.00	39/462	8.44
8. Sphenoid, lesser wing	0/18	0.00	1/38	2.63	25/178	14.04	9/117	7.69	0/36	0.00	35/387	9.04
9. Orbit, zygomatic internal	0/13	0.00	1/22	4.55	1/170	0.59	1/125	0.80	0/40	0.00	3/370	0.81
10. Infraorbital foramen, porosity	0/7	0.00	1/19	5.26	8/118	6.78	7/90	7.78	2/37	5.41	18/271	6.64
11. Infraorbital foramen, hyperostosis	0/7	0.00	0/19	0.00	0/118	0.00	1/90	1.11	0/37	0.00	1/271	0.37
12. Scapula, supraspinous	0/19	0.00	0/40	0.00	3/173	1.73	1/119	0.84	0/40	0.00	4/391	1.02
13. Femur, linea aspera	0/29	0.00	0/59	0.00	4/218	1.83	5/157	3.18	1/46	2.17	10/509	1.96
14. Tibia	2/27	7.41	3/49	6.12	20/205	9.76	30/148	20.27	8/43	18.60	63/472	13.35
Suggestive variables												
15. Cranial vault, porosity	1/27	3.70	4/57	7.02	4/210	1.90	4/139	2.88	1/41	2.44	14/474	2.95
16. Cranial vault, hyperostosis	1/27	3.70	4/57	7.02	4/210	1.90	0/139	0.00	1/41	2.44	10/474	2.11
17. Occipital, endocranial	4/23	17.39	11/55	20.00	71/218	32.57	28/142	19.72	9/44	20.45	123/482	25.52
18. Parietal, endocranial	1/27	3.70	6/57	10.53	30/210	14.29	10/139	7.19	3/41	7.32	50/474	10.55
19. Sphenoid, foramen rotundum	1/21	4.76	2/38	5.26	56/190	29.47	18/125	14.40	1/38	2.63	78/412	18.93
20. Orbit, porosity	0/25	0.00	1/55	1.82	48/209	22.97	55/140	39.29	14/42	33.33	118/471	25.05
21. Scapula, infraspinous	0/19	0.00	0/40	0.00	0/173	0.00	1/119	0.84	0/40	0.00	1/391	0.26
22. Humerus	3/30	10.00	3/59	5.08	4/225	1.78	9/156	5.77	5/47	10.64	24/517	4.64
23. Radius	1/29	3.45	0/44	0.00	2/208	0.96	0/155	0.00	1/46	2.17	4/482	0.83
24. Ulna	1/29	3.45	0/50	0.00	4/217	1.84	13/153	8.50	4/45	8.89	22/494	4.45
25. Coxae	0/26	0.00	0/45	0.00	3/218	1.38	4/154	2.60	3/45	6.67	10/488	2.05
26. Femur	2/29	6.90	0/58	0.00	14/221	6.33	20/158	12.66	6/47	12.77	42/513	8.19
27. Fibula	1/22	4.55	0/28	0.00	3/171	1.75	16/139	11.51	3/43	6.98	23/403	5.71

**TABLE 2:** Prevalence rates of scorbutic lesions in adult skeletons by age group

	18–25 years		26–35 years		36–45 years		≥ 46 years		> 18 years		All	
						
	No./Total	%	No./Total	%	No./Total	%	No./Total	%	No./Total	%	No./Total	%
Definite variables												
1. Sphenoid, greater wing	1/29	3.45	4/81	4.94	5/121	4.13	3/46	6.52	0/3	0.00	13/280	4.64
2. Maxilla, posterior	1/33	3.03	0/96	0.00	5/146	3.42	0/55	0.00	0/9	0.00	6/339	1.77
3. Mandible, medial coronoid	1/33	3.03	5/105	4.76	1/157	0.64	0/61	0.00	0/12	0.00	7/368	1.90
4. Palate, process	2/32	6.25	2/91	2.20	1/137	0.73	2/45	4.44	0/6	0.00	7/311	2.25
5. Maxilla, alveolar process	2/33	6.06	3/96	3.12	8/145	5.52	1/53	1.89	0/9	0.00	14/336	4.17
6. Mandible, alveolar process	1/33	3.03	3/107	2.80	2/158	1.27	2/62	3.23	0/12	0.00	8/372	2.15
Indicative variables												
7. Infraorbital foramen, porosity	0/30	0.00	4/75	5.33	3/106	2.83	1/30	3.33	0/3	0.00	8/244	3.28
8. Radius	2/33	5.71	5/109	4.59	5/159	3.14	5/63	7.94	0/16	0.00	17/382	4.45
9. Ulna	0/33	0.00	4/109	3.67	5/162	3.09	4/62	6.45	0/16	0.00	15/384	3.91
10. Coxae	3/33	9.09	4/92	4.35	2/152	1.32	3/54	5.56	0/3	0.00	12/334	3.59
11. Femur, diaphysis	7/35	20.00	10/108	9.26	6/162	3.70	5/61	8.20	2/24	8.33	30/390	7.69
12. Femur, linea aspera	6/35	17.14	6/108	5.56	1/162	0.62	4/61	6.56	0/24	0.00	17/390	4.36
13. Tibia	8/33	24.24	39/102	38.24	48/160	30.00	29/62	46.77	3/18	16.67	127/375	33.87
14. Fibula	6/34	17.65	15/99	15.15	20/148	13.51	13/63	20.63	3/13	23.08	57/357	15.97
15. Hum+Rad+Uln+Fem+Tib+Fib	2/33	6.06	2/91	2.20	0/141	0.00	1/59	1.69	0/5	0.00	5/329	1.52
16. Fem+Tib+Fib	5/33	15.15	7/98	7.14	4/147	2.72	7/62	11.29	0/12	0.00	23/352	6.53
Suggestive variables												
17. Parietal, endocranial	1/33	3.03	4/104	3.85	3/150	2.00	1/63	1.59	0/6	0.00	9/356	2.53
18. Sphenoid, lesser wing	0/29	0.00	1/83	1.20	1/123	0.81	0/43	0.00	0/4	0.00	2/282	0.71
19. Infraorbital foramen, hyperostosis	0/30	0.00	1/75	1.33	1/106	0.94	0/30	0.00	0/3	0.00	2/244	0.82
20. Scapula, supraspinous	0/33	0.00	2/90	2.22	0/143	0.00	0/53	0.00	0/3	0.00	2/322	0.62
21. Humerus	3/34	8.82	6/115	5.22	6/165	3.64	2/64	3.12	1/21	4.76	18/399	4.51
22. Femur	7/35	20.00	14/110	12.73	14/164	8.54	9/63	14.29	2/25	8.00	46/397	11.59

**Fig. 4:**
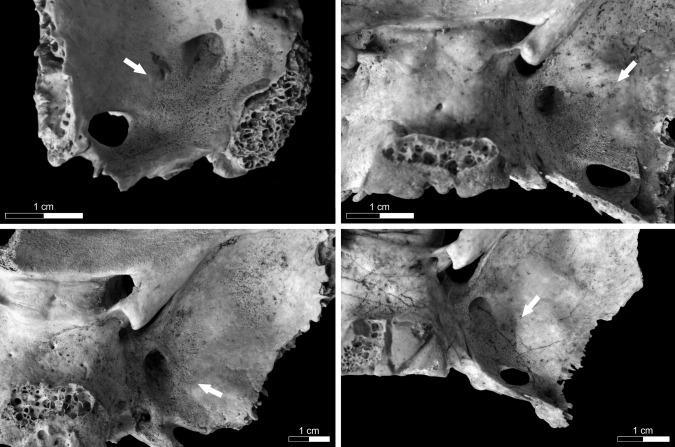
Examples of new bone formation around the foramen rotundum in a 3 year old child (upper left), 7–8 year old child (upper right), 5–6 year old child (lower left), and a 1–2 year old child (lower right).

Generalized nonspecific lesions, which correlated to at least one definite or one probable variable, were considered to be suggestive scorbutic lesions. In juveniles these comprised active porosity and hyperostosis of the cranial vault, endocranial lesions of the occipital and parietal bones, porous and hypertrophic lesions at the foramina rotundi of the sphenoid (Fig. 4), cribra orbitalia, periosteal lesions at the infraspinous area of the scapulae, and very fine proliferative new bone formation on the upper limb bones, the coxae, femora, and fibulae (see Table 1). In adults, the suggestive indicators consisted of active endocranial lesions of the parietals, porotic lesions of the lesser wings of the sphenoid, hyperostosis at the infraorbital foramina, periosteal lesions at the supraspinous area of the scapulae, and new bone formation of the humeri (see Fig. 3) and femora (see Table 2). Skeletons with at least one indicative or one suggestive scorbutic lesion were given a possible diagnosis of scurvy.

## RESULTS

### Population characteristics

Of the 970 skeletons, more than half (56.19%; *N* = 545) were of individuals who had died before the age of 18 years, with the largest age group comprising young children (24.64%; *N* = 239). Relatively few neonatal (3.30%; *N* = 32) and young adult (3.6%; *N* = 35) skeletons were present and, of the adults, the majority were middle adults (Table 3). The mean age-at-death, excluding adult skeletons that could not be aged precisely, was calculated as 19 years (SD = 17.47; *N* = 938).

**TABLE 3:** Demographic profile of the skeletal population from the Kilkenny Union Workhouse

Age group	Males	Females	Indet. sex	Total	%Total
< 1 month	–	–	32	32	3.30
1–12 months	–	–	63	63	6.49
1–5 years	–	–	239	239	24.64
6–12 years	–	–	163	163	16.80
13–17 years	4	3	41	48	4.95
18–25 years	18	17	0	35	3.61
26–35 years	46	74	1	121	12.47
36–45 years	94	74	4	172	17.73
≥ 46 years	44	20	1	65	6.70
> 18 years	10	12	10	32	3.30
All	216	200	554	970	100.00

Sex was determined for a total of 200 females and 216 males, seven of whom were adolescents. The mortality profile of the population, however, is expected to be influenced by the underlying demography of the workhouse. In general, workhouses only granted admission to orphans, complete families, or the very oldest and infirm members of society who had nobody else to care for them.

Estimated living stature in males ranged from 156 cm to 183 cm, with a mean height of 171 cm (SD = 5.41; *N* = 186), and in females from 146 cm to 179 cm with a mean height of 158 cm (SD = 5.59; *N* = 160). These estimations fit within the range of living stature calculations derived from 19th-century skeletal populations from Britain (Supporting Information Table S3), and would indicate that the adults at Kilkenny were not generally stunted due to previous poor health.

### Prevalence of scurvy

A definite diagnosis of scurvy was made in 16% (*N* = 156) of individuals, while probable scurvy was identified in 14% (*N* = 138) of the group, and possible scurvy was considered to be present in 21% (*N* = 205) of all assessable skeletons (*N* = 964), which gives an overall prevalence of approximately 52%. In the juvenile population, scorbutic lesions occurred in conjunction with rickets in 14 cases and with active tuberculosis in three cases. Among the adults diagnosed with scurvy, residual rickets were present in nine cases and active tuberculosis in one case.

#### Age in relation to scurvy

**Fig. 5:**
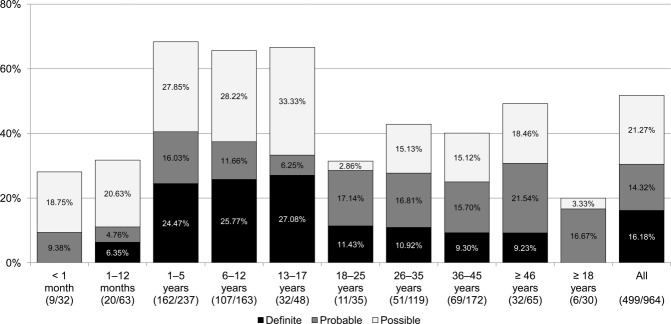
Prevalence rate of scorbutic skeletons with a definite, probable and possible diagnosis, by age.

When the data are analyzed by age (Fig. 5), the results indicate that the highest rates occurred among young children, older children, and adolescents, with total scurvy prevalences ranging from 66% to 68% ($ \bar x $
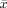
= 66.89%). Among the adults, the rates ranged from 31% in young adults to 49% in older adults ($ \bar x $
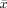
= 40.91%). The lowest frequencies were observed in neonatal and infant skeletons at 28% and 32%, respectively.

The age distribution of skeletons displaying scorbutic lesions versus unaffected skeletons differed, and is suggestive that scurvy influenced mortality in this population. The mean age-at-death of non-scorbutic skeletons was 22 years (SD = 18.02; *N* = 441), while the corresponding value for skeletons with scurvy was 17 years (SD = 16.62; *N* = 493). This difference was statistically significant (*t*(899) = 4.582, *P* < 0.001) for the overall population, but not for adults. Among adult females, the mean age-at-death was 37 years (SD = 8.14; *N* = 127) in non-scorbutic skeletons and 36 years (SD = 6.84; *N* = 57) in scorbutic skeletons (*t*(182) = 0.144, *P* = 0.886), while for the males, non-scorbutic skeletons displayed a mean age-at-death of 39 years (SD = 7.98; *N* = 96), compared with 40 years (SD = 7.98; *N* = 105) in scorbutic skeletons (*t*(199) = −0.362, *P* = 0.718).

**Fig. 6:**
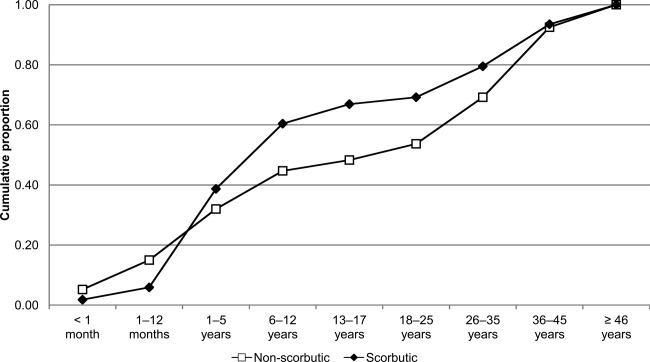
The cumulative age group distribution of scorbutic versus non-scorbutic skeletons.

A different overall age distribution was further confirmed in a two-sample Kolmogorov–Smirnov test (*z* = 2.844, *P* < 0.001), although this was only significant in juvenile age groups (*z* = 2.525, *P* < 0.001) and not for adults (*z* = 0.503, *P* = 0.962). A disparity in the mortality profile between scorbutic and non-scorbutic skeletons is, however, evident and illustrated by the cumulative age group distribution displayed in Figure 6. The highest risk of death, for those individuals who displayed scorbutic lesions, occurred between approximately 6 and 25 years of age. The greatest difference is noted among the adolescent population, where a 19% higher mortality rate is noted for skeletons with scurvy. The trend differs notably for the neonatal and infant age groups, for whom greater frequencies of individuals were identified as being non-scorbutic. This may be a reflection of the osteological paradox (see Wood et al.,^1992^), and it is possible that many of the youngest members of the population that suffered from scurvy had died before skeletal lesions had time to develop.

#### Sex in relation to scurvy

Among the adults, males were 1.7 times more frequently diagnosed with scurvy than females. This difference was statistically significant in the overall adult population, and also among the early middle adult and older adult components of the population. The largest difference was noted among the older adults, where over three times as many males compared to females displayed scorbutic lesions (Table 4). This discrepancy is probably a reflection of the difference in biological metabolism and daily Vitamin C intake requirement between the sexes (see above). It may indicate that the daily food ration supplied to the adult male population was inadequate (see Discussion below) and that they suffered a relatively greater loss of required nutritional intake than females (Carpenter,^1986^; Clarkson and Crawford,^2001^). The relief food portions depended on the age of the recipient—adults were given the largest portions, with children aged between 9 and 14 years having been given smaller portions, and the youngest children the least (O'Connor,^1995^). None of the available sources for the Kilkenny Union Workhouse suggest that males and females were given portions of different size, but information derived from other workhouses would tend to suggest that sex differentiation of this nature frequently occurred (see Clarkson and Crawford,^2001^).

**TABLE 4:** Differences in diagnosed scurvy frequencies between adult males and females by age group and chi-square test values (df = 1)

	Males		Females			
				
Age group	No./Total	%	No./Total	%	χ^2^	*P*-value
18–25 years	8/18	44.44	3/17	17.65	2.913	0.088
26–35 years	26/45	57.78	25/73	34.25	6.281	0.012
36–45 years	43/94	45.74	25/74	33.78	2.459	0.117
≥46 years	28/44	63.64	4/20	20.00	10.473	0.001
>18 years	2/9	22.22	1/12	8.33	0.810	0.368
All	107/210	50.94	58/196	29.59	19.175	< 0.001

#### Size in relation to scurvy

Physical size appears to be a differentiating factor between scorbutic and non-scorbutic skeletons in the adult male population (Table 5). The mean estimated living stature for non-scorbutic males was 170 cm as opposed to 172 cm for scorbutic skeletons, and was statistically significant. This finding may indicate that size was a contributing factor as to why males displayed more scorbutic skeletal lesions than females. In addition, it would also tend to support the assertion that the relief food supplied to males in Kilkenny was inadequate in terms of their nutritional requirements. It is unlikely that well-built individuals would have been provided with additional food rations, and they would therefore have been deprived of a relatively higher proportion of nutrients compared to smaller individuals. An anthropometric study of the prefamine and postfamine population of Ukraine in the 1920s demonstrated that starvation had a greater impact on taller individuals (Ivanovsky,^1923^), although this trend was not observed in the Minnesota Experiment of the 1940s, during which the physiological and mental effects of starvation were investigated (Keys et al.,^1950^).

**TABLE 5:** Differences in stature between non-scorbutic and scorbutic adult skeletons by sex and in total

	Non-scorbutic					Scorbutic							
					
	Min.	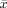	Max.	SD	*N*	Min.	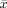	Max.	SD	*N*	*t*-value	df	*P*-value
Males													
Estimated living stature (cm)	155.55	170.43	183.04	5.18	86	160.46	172.23	183.15	5.48	100	−2.290	184	0.023
Femur greatest length (mm)	388.00	448.33	507.00	23.58	60	415.00	460.81	507.00	23.85	73	−3.017	131	0.003
Females													
Estimated living stature (cm)	147.22	157.88	178.64	5.26	109	146.11	159.00	171.43	6.30	50	−1.176	157	0.241
Femur greatest length (mm)	377.00	417.46	461.00	18.89	80	373.00	423.74	475.00	25.07	43	−1.438	68	0.155
All													
Estimated living stature (cm)	147.22	163.41	183.04	8.13	195	146.11	167.83	183.15	8.47	151	−4.925	344	<0.001
Femur greatest length (mm)	377.00	430.69	507.00	25.97	140	373.00	447.07	507.00	30.15	116	−4.669	254	<0.001

## DISCUSSION

When the results from Kilkenny are compared to frequencies obtained for other macroscopically analyzed skeletal populations, which have ranged from approximately 3–7% (see Brickley and Ives,^2008^: Table A1), it is clear that the prevalence of scurvy in the Kilkenny Workhouse Union population is exceptionally high.

As scorbutic skeletal lesions only appear after a re-introduction of Vitamin C, the skeletons with signs of scurvy in the Kilkenny Union Workhouse population are therefore likely to have been individuals who had been deprived of Vitamin C as a result of the potato blight, but were then provided with the vitamin in the workhouse diet once they had been admitted to the institution. It may also be the case that scorbutic skeletons represent those individuals who had resided for longer in the workhouse prior to death than those who did not display skeletal evidence of the disease. The non-scorbutic skeletons could thus have been individuals who died before any osseous changes had commenced, even though they had probably been given some access to Vitamin C once they had entered the workhouse.

Diet in the Kilkenny workhouse would have varied throughout the famine years depending on economic circumstances. During the worst of the crisis, it essentially comprised only bread and water. In December 1848, the adult diet was reported as consisting of eight ounces (227 g) of Indian meal (maize), one ounce (28 g) of rice and ½ pint (300 ml) of new milk for breakfast, and ¾ pound (340 g) of brown bread and ½ pint of a vegetable soup made of rice, oatmeal, turnips, parsnips, and onion for dinner (Anon,^1848^). Some Vitamin C would evidently have been acquired from the vegetables and milk of this diet.

Despite the high prevalence of skeletal scurvy in the Kilkenny Union Workhouse population, there is a noticeable absence to a reference of any kind to the disease in the workhouse's original minute books or within contemporary articles in local newspapers. None of the surviving reports written by the workhouse physicians make any mention of the disease and there are strong reasons to believe it had been misdiagnosed. In 1846 at the Ballymore Eustace Dispensary in Naas, severely ill patients were initially described as suffering from “a peculiar form” of gastroenteritis before their condition was correctly diagnosed as scurvy (Crawford,^1988^: 286). Similarly, during the widespread occurrence of scurvy in Bath, Somerset, in England during 1847, workhouse physicians incorrectly diagnosed cases of scurvy as purpura hemorrhagica (Barrett,^1849^).

It is within this context, that an article published by Dr. Joseph Lalor in *The Dublin Quarterly Journal of Medical Science* in 1848 should be understood (Lalor,^1848^). Lalor was one of the residing physicians in the Kilkenny workhouse and he described in great detail the nature of an epidemic that took place in Kilkenny between 1844 and 1847, which he diagnosed as “gastric fever,” which implies typhoid fever. It is clear from his text that some of the symptoms are in fact classic characteristics of scurvy. From the summer of 1845 he had noted “purpuric eruptions” in his patients, and these are likely to represent scorbutic ecchymoses:

“The order of frequency in which the skin of the different parts of the body was the seat of eruption, was pretty much as follows: the upper part of the chest, the inguinal regions, the back of the neck and throat, the abdomen, the flexures of the elbow-joints, the arms, fore-arms, legs, thighs, lumbar region, and lower part of chest. […] [T]he persistence of the spots after pressure by the finger was sufficient to distinguish them from the spots of typhus” (Lalor,^1848^: 16–17).

Lalor's description of the distribution of bruises has some similarities to the distribution of periosteal lesion in the Kilkenny skeletal population, particularly those at the elbows (distal humerus) and the lower arm and lower limb bones (see Tables 1 and 2). Lalor probably believed that the purpura was related to the reigning famine fever but the exceptionally high prevalence of scurvy identified in the skeletons from the Kilkenny Union Workhouse would tend to suggest that a misdiagnosis of scurvy had been made by the workhouse physicians during the famine.

Although the influence of Vitamin C on the immune system is not yet fully understood, experiments have shown how ascorbic acid influences the neutrophil chemotaxis and acts as an immunostimulant. This occurs through an increase in T and B lymphocyte proliferation and as a result of migrating cells reaching sites of infection. Viruses and bacteria are also more susceptible to destruction as a result of the influence of ascorbic acid (Anderson,^1982^; Hemilä,^2003^; Ronzio^2003^). As discussed above, scurvy appears to have significantly influenced the mortality pattern in the Kilkenny Union Workhouse population and it seems likely that this impairment of the immune system was a contributing risk factor in the acquisition of infectious diseases such as measles, scarlet fever, typhus, relapsing fever, smallpox, tuberculosis, and cholera (see Geary,^1996^; Patterson,^1997^; Kennedy et al.,^1999^; Mokyr and Ó Gráda,^1999^) which, for many people at that time, resulted in death.

A lesion of particular interest in the Kilkenny skeletal population, which is labeled as a suggestive indicator of scurvy, was well defined porous and proliferative new bone formation localized around the foramina rotundi of the sphenoid (see Fig. 4). The authors have only found one published reference which discusses these lesions in relation to Vitamin C deficiency (see Brown and Ortner,^2011^). In the Kilkenny population, they were prevalent in 19% of all juveniles (Table 1). When only scorbutic juveniles are considered the lesion was found to occur in 27% of all scorbutic skeletons—19% (20/108) of cases of definite scurvy and 51% (40/78) of cases of probable scurvy. The foramina rotundi lesions were only present in four adults and did not statistically correlate with any other scorbutic lesions. Their occurrence in juveniles, with both definite and probable scorbutic lesions, appears to verify their association with Vitamin C deficiency. The osseous lesions noted at the foramina rotundi were virtually identical to the inflammatory reaction observed around the infraorbital foramina, which has previously been described as potentially relating to scurvy (Ortner and Erickson,^1997^; Ortner et al.,^2001^). Considering that both the infraorbital foramen and the foramen rotundum facilitate the same maxillary nerve and artery it appears likely that the inflammatory osseous reaction noted at both of these landmarks has a common etiological factor, namely hemorrhage.

Hemorrhages of nerves and blood vessels were observed and described in an early twentieth-century study of fallen soldiers from World War I who were known to have suffered from scurvy (Aschoff and Koch,^1919^). Bleeding of the nerve tracts in relation to scurvy have also been mentioned elsewhere (e.g., Hodges et al.,^1969^; Friedrich,^1988^), although no specific reference to the maxillary nerve has been made. Equally, deficiency of Vitamin C can result in arterial hemorrhage as impaired collagen regeneration will eventually cause the arterial walls to rupture. A ruptured maxillary artery is an unlikely primary cause of death, but it may have been responsible for the porous new bone formation secondary to localized hemorrhage noted at both the foramina rotundi and the infraorbital foramina in the skeletal population from the Kilkenny Union Workhouse.

## CONCLUSIONS

The paleopathological evidence of scurvy in the Kilkenny Union Workhouse skeletal population has provided the first opportunity to study the nature of the disease in a known archaeological famine population. Scurvy occurred with an exceptionally high prevalence and it appears to have had a significant impact on the mortality profile of the population. This may have arisen indirectly through a suppression of the immune system which would have made sufferers more prone to the acquisition of infectious diseases which inevitably resulted in their death. A comparison of the paleopathological evidence with information contained within historical and archival sources for the Kilkenny Union Workhouse indicates that scurvy was very probably misdiagnosed by the workhouse physicians during the famine.

Scurvy is potentially a severely painful disease and these unfortunate people would undoubtedly have been subjected to arduous and repetitive labor during their time in the workhouse. As such, it can be concluded, on the basis of the paleopathological analysis, that many of them would have endured a very painful existence during the last weeks or months of their lives. Clinical research has also indicated that Vitamin C deficiency can have a negative effect on a person's mental wellbeing and can lead to the development of depression and apathy (Kinsman and Hood,^1971^; Dixit,^1979^). The human experience of the Great Irish Famine was not just starvation and disease. It would also have been felt on a deeply emotional level (see Paleologos et al.,^1998^; Bowman et al.,^2009^) and resulted in consequences which persisted for an entire generation afterwards and beyond (see Galler and Barrett,^2001^).
