# Supplementary material for: Scurvy in the Great Irish Famine: Evidence of Vitamin C Deficiency From a Mid-19th Century Skeletal Population
Source: Am J Phys Anthropol. 2012 Mar 28;148(4):512–24. doi: 10.1002/ajpa.22066 (PMC3467765; doi:10.1002/ajpa.22066)
Supplement: Supplementary file 1 [file ajpa0148-0512-SD1.docx]

TABLE S1. Two-tailed Pearson’s correlation test values of scorbutic lesions in juvenile skeletons. Explanations of each numbered variable are provided in Table 1. Significant correlations are marked bold for the 0.05 level and bold and italics for the 0.01 level.

|  | 1 | 2 | 3 | 4 | 5 | 6 | 7 | 8 | 9 | 10 | 11 | 12 | 13 | 14 | 15 | 16 | 17 | 18 | 19 | 20 | 21 | 22 | 23 | 24 | 25 | 26 | 27 |
| --- | --- | --- | --- | --- | --- | --- | --- | --- | --- | --- | --- | --- | --- | --- | --- | --- | --- | --- | --- | --- | --- | --- | --- | --- | --- | --- | --- |
| 1 | 1 |  |  |  |  |  |  |  |  |  |  |  |  |  |  |  |  |  |  |  |  |  |  |  |  |  |  |
| 2 | -0,030  (345) | 1 |  |  |  |  |  |  |  |  |  |  |  |  |  |  |  |  |  |  |  |  |  |  |  |  |  |
| 3 | 0.057  (379) | 0.094  (404) | 1 |  |  |  |  |  |  |  |  |  |  |  |  |  |  |  |  |  |  |  |  |  |  |  |  |
| 4 | 0.060  (322) | ***0.285***  ***(371)*** | 0.064  (369) | 1 |  |  |  |  |  |  |  |  |  |  |  |  |  |  |  |  |  |  |  |  |  |  |  |
| 5 | 0.010  (344) | ***0.140***  ***(404)*** | ***0.185***  ***(402)*** | ***0.387***  ***(375)*** | 1 |  |  |  |  |  |  |  |  |  |  |  |  |  |  |  |  |  |  |  |  |  |  |
| 6 | **0.109**  **(380)** | ***0.166***  ***(406)*** | ***0.237***  ***(485)*** | ***0.139***  ***(370)*** | ***0.346***  ***(404)*** | 1 |  |  |  |  |  |  |  |  |  |  |  |  |  |  |  |  |  |  |  |  |  |
| 7 | ***0.146***  ***(375)*** | 0.016  (386) | -0.021  (441) | **0.109**  **(351)** | -0.072  (384) | -0.029  (448) | 1 |  |  |  |  |  |  |  |  |  |  |  |  |  |  |  |  |  |  |  |  |
| 8 | **0.131**  **(354)** | 0.003  (349) | 0.038  (378) | ***0.158***  ***(322)*** | -0.082  (348) | 0.022  (381) | ***0.242***  ***(376)*** | 1 |  |  |  |  |  |  |  |  |  |  |  |  |  |  |  |  |  |  |  |
| 9 | ***0.261***  ***(314)*** | -0.020  (340) | 0.062  (362) | ***0.203***  ***(316)*** | -0.019  (340) | 0.077  (364) | 0.085  (351) | ***0.184***  ***(317)*** | 1 |  |  |  |  |  |  |  |  |  |  |  |  |  |  |  |  |  |  |
| 10 | 0.051  (249) | **0.147**  **(266)** | ***0.182***  ***(267)*** | ***0.227***  ***(270)*** | **0.151**  **(271)** | ***0.242***  ***(268)*** | 0.045  (261) | 0.090  (244) | ***0.227***  ***(241)*** | 1 |  |  |  |  |  |  |  |  |  |  |  |  |  |  |  |  |  |
| 11 | -0.019  (249) | ***0.228***  ***(266)*** | ***0.169***  ***(267)*** | -0.014  (270) | -0.017  (271) | ***0.176***  ***(268)*** | -0.017  (261) | -0.020  (244) | -0.004  (241) | ***0.228***  ***(271)*** | 1 |  |  |  |  |  |  |  |  |  |  |  |  |  |  |  |  |
| 12 | -0.028  (326) | -0.026  (345) | **0.123**  **(374)** | ***0.151***  ***(320)*** | 0.089  (342) | 0.060  (379) | 0.079  (362) | 0.071  (328) | -0.005  (312) | -0.017  (246) | -0.004  (246) | 1 |  |  |  |  |  |  |  |  |  |  |  |  |  |  |  |
| 13 | 0.010  (374) | **0.102**  **(398)** | 0.047  (463) | 0.065  (363) | ***0.172***  ***(396)*** | 0.065  (471) | -0.046  (439) | -0.050  (374) | -0.015  (362) | -0.048  (264) | -0.011  (264) | -0.015  (381) | 1 |  |  |  |  |  |  |  |  |  |  |  |  |  |  |
| 14 | ***0.154***  ***(359)*** | 0.065  (380) | 0.020  (434) | ***0.153***  ***(346)*** | 0.098  (379) | 0.000  (441) | ***0.129***  ***(419)*** | 0.063  (360) | 0.078  (346) | 0.108  (254) | -0.026  (254) | -0.041  (370) | ***0.201***  ***(467)*** | 1 |  |  |  |  |  |  |  |  |  |  |  |  |  |
| 15 | 0.014  (376) | 0.028  (392) | -0.015  (448) | 0.072  (357) | 0.033  (389) | **0.096**  **(455)** | ***0.150***  ***(439)*** | ***0.162***  ***(377)*** | ***0.181***  ***(354)*** | 0.064  (264) | -0.009  (264) | ***0.181***  ***(371)*** | 0.060  (449) | ***0.137***  ***(429)*** | 1 |  |  |  |  |  |  |  |  |  |  |  |  |
| 16 | 0.043  (376) | -0.032  (392) | 0.004  (448) | **0.104**  **(357)** | -0.032  (389) | 0.075  (455) | ***0.204***  ***(439)*** | ***0.227***  ***(377)*** | ***0.227***  ***(354)*** | 0.117  (264) | -0.007  (264) | ***0.227***  ***(371)*** | -0.023  (449) | 0.045  (429) | ***0.842***  ***(474)*** | 1 |  |  |  |  |  |  |  |  |  |  |  |
| 17 | 0.029  (379) | -0.013  (391) | 0.059  (453) | 0.023  (358) | 0.008  (387) | 0.024  (459) | ***0.324***  ***(441)*** | ***0.296***  ***(377)*** | 0.090  (359) | 0.048  (265) | -0.033  (265) | -0.086  (373) | -0.017  (460) | 0.023  (431) | ***0.132***  ***(455)*** | ***0.121***  ***(455)*** | 1 |  |  |  |  |  |  |  |  |  |  |
| 18 | 0.094  (376) | -0.013  (392) | 0.076  (448) | 0.024  (357) | 0.020  (389) | 0.032  (455) | ***0.505***  ***(439)*** | ***0.254***  ***(377)*** | ***0.252***  ***(354)*** | 0.013  (264) | -0.021  (264) | 0.033  (371) | -0.003  (449) | ***0.146***  ***(429)*** | 0.062  (474) | **0.093**  **(474)** | ***0.313***  ***(455)*** | 1 |  |  |  |  |  |  |  |  |  |
| 19 | -0.042  (385) | -0.008  (366) | 0.002  (403) | 0.065  (338) | -0.064  (363) | 0.027  (404) | ***0.292***  ***(398)*** | ***0.473***  ***(372)*** | 0.031  (332) | 0.080  (255) | -0.032  (255) | 0.030  (345) | 0.037  (297) | **0.109**  **(382)** | 0.078  (401) | 0.084  (401) | ***0.361***  ***(403)*** | ***0.306***  ***(401)*** | 1 |  |  |  |  |  |  |  |  |
| 20 | 0.072  (375) | 0.049  (386) | **0.094**  **(448)** | 0.004  (352) | 0.017  (383) | 0.071  (454) | -0.032  (455) | 0.034  (376) | 0.008  (353) | 0.039  (260) | 0.086  (260) | 0.007  (364) | 0.015  (447) | 0.027  (422) | -0.012  (445) | -0.049  (445) | 0.017  (447) | 0.012  (445) | 0.035  (398) | 1 |  |  |  |  |  |  |  |
| 21 | n/a  (326) | -0.013  (345) | -0.019  (374) | -0.011  (320) | -0.013  (342) | -0.016  (379) | ***0.169***  ***(362)*** | ***0.160***  ***(328)*** | -0.005  (312) | n/a  (246) | n/a  (246) | -0.005  (391) | -0.008  (381) | **0.132**  **(370)** | -0.008  (371) | -0.007  (371) | 0.089  (373) | ***0.144***  ***(371)*** | **0.107**  **(345)** | -0.034  (364) | 1 |  |  |  |  |  |  |
| 22 | 0.084  (379) | 0.053  (399) | 0.056  (471) | 0.031  (364) | -0.051  (396) | 0.045  (480) | ***0.171***  ***(450)*** | 0.063  (380) | ***0.268***  ***(363)*** | -0.014  (266) | -0.013  (266) | -0.023  (386) | 0.036  (490) | ***0.402***  ***(459)*** | 0.023  (458) | 0.042  (458) | 0.047  (467) | ***0.153***  ***(458)*** | 0.007  (403) | -0.006  (457) | ***0.224***  ***(386)*** | 1 |  |  |  |  |  |
| 23 | -0.027  (367) | 0.085  (386) | 0.033  (447) | 0.021  (356) | -0.025  (385) | 0.058  (455) | 0.077  (428) | 0.075  (367) | -0.008  (357) | -0.023  (260) | -0.005  (260) | -0.009  (369) | -0.014  (465) | ***0.175***  ***(440)*** | -0.017  (431) | -0.014  (431) | ***0.145***  ***(436)*** | ***0.199***  ***(431)*** | 0.079  (390) | -0.049  (432) | -0.005  (369) | ***0.301***  ***(475)*** | 1 |  |  |  |  |
| 24 | **0.132**  **(372)** | -0.006  (394) | 0.029  (460) | 0.098  (362) | 0.000  (392) | 0.013  (469) | 0.093  (435) | 0.062  (372) | **0.119**  **(361)** | 0.008  (263) | -0.014  (263) | -0.024  (377) | -0.040  (476) | ***0.435***  ***(446)*** | -0.087  (442) | -0.043  (442) | 0.022  (449) | ***0.158***  ***(442)*** | 0.063  (395) | **0.097**  **(442)** | ***0.224***  ***(377)*** | ***0.544***  ***(485)*** | ***0.418***  ***(472)*** | 1 |  |  |  |
| 25 | 0.089  (372) | **0.111**  **(394)** | 0.052  (453) | 0.027  (359) | 0.037  (392) | 0.064  (460) | ***0.126***  ***(431)*** | 0.009  (370) | -0.015  (360) | -0.041  (263) | -0.009  (263) | -0.013  (382) | ***0.183***  ***(474)*** | ***0.202***  ***(447)*** | 0.068  (438) | -0.021  (438) | **0.120**  **(446)** | 0.089  (438) | 0.012  (392) | -0.050  (436) | -0.006  (382) | ***0.182***  ***(471)*** | ***0.146***  ***(453)*** | 0.107  (465) | 1 |  |  |
| 26 | 0.074  (375) | 0.074  (400) | 0.019  (466) | 0.097  (364) | 0.080  (398) | 0.047  (474) | 0.083  (443) | 0.023  (375) | 0.067  (364) | 0.019  (264) | -0.019  (264) | -0.032  (382) | ***0.472***  ***(507)*** | ***0.533***  ***(469)*** | 0.079  (451) | -0.047  (451) | 0.016  (460) | **0.095**  **(451)** | 0.008  (399) | 0.000  (450) | 0.164  (382) | ***0.386***  ***(494)*** | ***0.296***  ***(468)*** | ***0.402***  ***(478)*** | ***0.316***  ***(479)*** | 1 |  |
| 27 | 0.107  (326) | 0.037  (345) | 0.021  (381) | 0.012  (318) | 0.041  (346) | -0.041  (386) | ***0.137***  ***(367)*** | -0.038  (324) | -0.021  (327) | -0.010  (237) | -0.018  (237) | -0.020  (334) | ***0.304***  ***(400)*** | ***0.503***  ***(399)*** | **0.105**  **(372)** | -0.030  (372) | 0.054  (372) | 0.055  (372) | 0.025  (347) | 0.040  (365) | 0.217  (334) | ***0.436***  ***(394)*** | ***0.191***  ***(391)*** | ***0.615***  ***(391)*** | ***0.166***  ***(391)*** | ***0.483***  ***(402)*** | 1 |

TABLE S2. Two-tailed Pearson’s correlation test values of scorbutic lesions in adult skeletons. Explanations of each numbered variable are provided in Table 2. Significant correlations are marked bold for the 0.05 level and bold and italics for the 0.01 level.

|  | 1 | 2 | 3 | 4 | 5 | 6 | 7 | 8 | 9 | 10 | 11 | 12 | 13 | 14 | 15 | 16 | 17 | 18 | 19 | 20 | 21 | 22 |
| --- | --- | --- | --- | --- | --- | --- | --- | --- | --- | --- | --- | --- | --- | --- | --- | --- | --- | --- | --- | --- | --- | --- |
| 1 | 1 |  |  |  |  |  |  |  |  |  |  |  |  |  |  |  |  |  |  |  |  |  |
| 2 | 0.083  (269) | 1 |  |  |  |  |  |  |  |  |  |  |  |  |  |  |  |  |  |  |  |  |
| 3 | -0.032  (271) | -0.020  (330) | 1 |  |  |  |  |  |  |  |  |  |  |  |  |  |  |  |  |  |  |  |
| 4 | -0.037  (255) | **0.136**  **(311)** | **0.269**  **(304)** | 1 |  |  |  |  |  |  |  |  |  |  |  |  |  |  |  |  |  |  |
| 5 | -0.051  (267) | ***0.197***  ***(336)*** | 0.073  (327) | ***0.490***  ***(311)*** | 1 |  |  |  |  |  |  |  |  |  |  |  |  |  |  |  |  |  |
| 6 | -0.035  (271) | -0.021  (331) | ***0.252***  ***(368)*** | ***0.386***  ***(305)*** | ***0.358***  ***(328)*** | 1 |  |  |  |  |  |  |  |  |  |  |  |  |  |  |  |  |
| 7 | 0.093  (216) | -0.029  (244) | 0.119  (238) | ***0.268***  ***(243)*** | 0.064  (243) | ***0.243***  ***(238)*** | 1 |  |  |  |  |  |  |  |  |  |  |  |  |  |  |  |
| 8 | -0.048  (271) | -0.033  (322) | 0.062  (347) | ***0.175***  ***(299)*** | 0.017  (319) | ***0.158***  ***(349)*** | ***0.193***  ***(239)*** | 1 |  |  |  |  |  |  |  |  |  |  |  |  |  |  |
| 9 | -0.041  (273) | 0.084  (326) | 0.081  (350) | ***0.335***  ***(301)*** | 0.104  (323) | ***0.171***  ***(351)*** | ***0.226***  ***(240)*** | ***0.806***  ***(376)*** | 1 |  |  |  |  |  |  |  |  |  |  |  |  |  |
| 10 | -0.046  (261) | -0.028  (295) | -0.030  (314) | ***0.215***  ***(276)*** | **0.124**  **(293)** | 0.083  (317) | 0.084  (231) | ***0.376***  ***(325)*** | ***0.415***  ***(325)*** | 1 |  |  |  |  |  |  |  |  |  |  |  |  |
| 11 | -0.065  (272) | 0.043  (317) | **0.118**  **(343)** | **0.121**  **(294)** | -0.009  (314) | ***0.193***  ***(346)*** | 0.118  (241) | ***0.592***  ***(365)*** | ***0.459***  ***(364)*** | ***0.265***  ***(330)*** | 1 |  |  |  |  |  |  |  |  |  |  |  |
| 12 | -0.048  (272) | 0.083  (317) | 0.074  (343) | ***0.183***  ***(294)*** | 0.028  (314) | ***0.272***  ***(346)*** | ***0.181***  ***(241)*** | ***0.461***  ***(365)*** | ***0.347***  ***(364)*** | ***0.294***  ***(330)*** | ***0.740***  ***(390)*** | 1 |  |  |  |  |  |  |  |  |  |  |
| 13 | 0.002  (273) | -0.004  (319) | 0.071  (340) | **0.123**  **(297)** | 0.036  (316) | **0.136**  **(342)** | -0.039  (240) | ***0.264***  ***(350)*** | ***0.237***  ***(351)*** | ***0.171***  ***(321)*** | ***0.283***  ***(366)*** | ***0.268***  ***(366)*** | 1 |  |  |  |  |  |  |  |  |  |
| 14 | -0.054  (270) | 0.003  (309) | ***0.193***  ***(329)*** | 0.071  (287) | -0.004  (306) | **0.112**  **(331)** | 0.050  (232) | ***0.454***  ***(338)*** | ***0.396***  ***(338)*** | ***0.235***  ***(314)*** | ***0.433***  ***(349)*** | ***0.422***  ***(349)*** | ***0.545***  ***(354)*** | 1 |  |  |  |  |  |  |  |  |
| 15 | -0.025  (260) | -0.019  (296) | -0.018  (313) | ***0.190***  ***(278)*** | 0.100  (293) | ***0.168***  ***(313)*** | ***0.170***  ***(229)*** | ***0.549***  ***(329)*** | ***0.589***  ***(329)*** | ***0.322***  ***(304)*** | ***0.424***  ***(328)*** | ***0.330***  ***(328)*** | ***0.172***  ***(329)*** | ***0.287***  ***(328)*** | 1 |  |  |  |  |  |  |  |
| 16 | -0.061  (268) | 0.052  (307) | ***0.145***  ***(326)*** | **0.146**  **(287)** | 0.004  (304) | **0.213**  **(328)** | **0.131**  **(233)** | ***0.548***  ***(334)*** | ***0.474***  ***(335)*** | ***0.279***  ***(312)*** | ***0.687***  ***(347)*** | ***0.627***  ***(347)*** | ***0.361***  ***(352)*** | ***0.621***  ***(351)*** | ***0.453***  ***(329)*** | 1 |  |  |  |  |  |  |
| 17 | 0.055  (276) | -0.023  (322) | -0.021  (342) | 0.102  (297) | 0.061  (319) | -0.021  (344) | -0.037  (239) | 0.046  (335) | 0.053  (336) | 0.070  (308) | ***0.159***  ***(336)*** | 0.053  (336) | **0.188**  **(334** | ***0.179***  ***(326)*** | **0.130**  **(310)** | 0.099  (323) | 1 |  |  |  |  |  |
| 18 | -0.019  (267) | -0.013  (273) | -0.013  (273) | n/a  (259) | -0.019  (271) | -0.014  (273) | n/a  (218) | **0.191**  **(272)** | -0.016  (275) | -0.012  (261) | -0.017  (275) | -0.012  (275) | 0.111  (275) | 0.077  (272) | -0.008  (261) | -0.016  (270) | -0.016  (278) | 1 |  |  |  |  |
| 19 | -0.020  (216) | -0.014  (244) | -0.015  (238) | -0.014  (243) | -0.021  (243) | -0.016  (238) | ***0.494***  ***(244)*** | -0.019  (239) | -0.018  (240) | -0.019  (231) | -0.027  (241) | -0.020  (241) | -0.067  (240) | -0.043  (232) | -0.013  (229) | -0.028  (233) | -0.018  (239) | n/a  (218) | 1 |  |  |  |
| 20 | ***0.271***  ***(262)*** | -0.012  (295) | -0.012  (308) | -0.010  (277) | -0.019  (293) | -0.012  (308) | -0.013  (228) | ***0.177***  ***(313)*** | ***0.193***  ***(315)*** | -0.011  (294) | **0.124**  **(312)** | -0.017  (312) | 0.108  (313) | 0.076  (310) | ***0.348***  ***(297)*** | **0.138**  **(305)** | **0.226**  **(307)** | -0.008  (264) | -0.006  (228) | 1 |  |  |
| 21 | 0.043  (279) | -0.030  (336) | -0.031  (363) | 0.082  (310) | 0.023  (333) | 0.056  (365) | 0.095  (243) | **0.551**  **(376)** | ***0.479***  ***(378)*** | ***0.210***  ***(326)*** | ***0.377***  ***(372)*** | ***0.334***  ***(372)*** | ***0.213***  ***(360)*** | ***0.218***  ***(343)*** | ***0.549***  ***(329)*** | ***0.260***  ***(339)*** | **0.131**  **(350)** | ***0.212***  ***(281)*** | -0.017  (243) | ***0.371***  ***(320)*** | 1 |  |
| 22 | -0.082  (277) | 0.019  (323) | 0.077  (348) | 0.079  (299) | -0.033  (320) | **0.123**  **(351)** | 0.067  (242) | ***0.510***  ***(368)*** | ***0.395***  ***(367)*** | ***0.285***  ***(331)*** | ***0.789***  ***(390)*** | ***0.584***  ***(390)*** | ***0.361***  ***(371)*** | ***0.412***  ***(354)*** | ***0.334***  ***(329)*** | ***0.700***  ***(352)*** | **0.108**  **(340)** | -0.022  (279) | -0.035  (242) | 0.090  (317) | ***0.364***  ***(378)*** | 1 |

TABLE S3. Living stature estimations (cm) of the Kilkenny Union Workhouse population and contemporaneous skeletal populations from Britain, estimated using the methods of Trotter and Gleser (1952; 1958).

| Population | Date | Males | | | | |  | Females | | | | |
| --- | --- | --- | --- | --- | --- | --- | --- | --- | --- | --- | --- | --- |
|  |  | *Min.* | $\bar{x}$ | *Max.* | *SD* | *N* |  | *Min.* | $\bar{x}$ | *Max.* | *SD* | *N* |
| Kilkenny Union Workhouse, Kilkenny City | 1847–51 | 155.55 | 171.40 | 183.15 | 5.41 | 186 |  | 146.11 | 158.22 | 178.64 | 5.59 | 160 |
| Cross Bones, Southwark, London^a^ | c. 1820–80 | 153.00 | 168.50 | 180.00 | n/a | 16 |  | 142.00 | 158.20 | 172.00 | n/a | 19 |
| Spitalfields, London^b^ | 1729–1852 | 154.62 | 170.27 | 187.79 | 6.16 | 211 |  | 139.81 | 157.11 | 173.65 | 6.25 | 206 |
| St Peter's Collegiate Church, Wolverhampton^c^ | c. 1840–70 | 161.70 | 171.00 | 181.30 | 5.37 | 30 |  | 150.40 | 160.60 | 173.00 | 5.20 | 25 |
| St Luke's Church, Islington, London^d^ | 1760–1850 | 149.00 | 171.00 | 194.00 | n/a | 295 |  | 139.00 | 158.00 | 174.00 | n/a | 238 |
| St Marylebone Church, London^e^ | 1767–1859 | 154.00 | 170.00 | 182.00 | 5.61 | 76 |  | 145.00 | 159.00 | 169.00 | 5.44 | 62 |
| St Martin's-in-the-Bull Ring, Birmingham^f^ | c. 1775–1863 | 156.00 | 171.90 | 185.00 | 5.59 | 173 |  | 139.00 | 159.10 | 170.50 | 5.65 | 124 |
| St Peter's, Barton-upon-Humber^g^ | 1700–1855 | 158.00 | 171.00 | 185.00 | n/a | n/a |  | 147.00 | 159.00 | 170.00 | n/a | n/a |
| ^a^ Brickley et al. (1999)  ^b^ Molleson et al. (1993)  ^c^ Arabaolaza et al. (2007)  ^d^ Boyle et al. (2005)  ^e^ Miles et al. (2008)  ^f^ Brickley et al. (2006)  ^g^ Waldron (2007b) | | | | | | | | | | | | |
